# Exercise-induced central and peripheral sympathetic activity in a community-based group of epilepsy patients differ from healthy controls

**DOI:** 10.1007/s00221-024-06792-0

**Published:** 2024-03-29

**Authors:** Franziska van den Bongard, Julia Kristin Gowik, Jessica Coenen, Rasmus Jakobsmeyer, Claus Reinsberger

**Affiliations:** 1 Institute of Sports Medicine, Paderborn University, Warburger Straße 100, 33098 Paderborn, Germany; 2 Division of Sports Neurology & Neurosciences, Department of Neurology, Mass General Brigham, Harvard Medical School, Boston, MA USA

**Keywords:** Autonomic nervous system, Functional connectivity, Exhaustive exercise, Seizure-free, Epilepsy

## Abstract

Ictal and interictal activity within the autonomic nervous system is characterized by a sympathetic overshoot in people with epilepsy. This autonomic dysfunction is assumed to be driven by alterations in the central autonomic network. In this study, exercise-induced changes of the interrelation of central and peripheral autonomic activity in patients with epilepsy was assessed. 21 patients with epilepsy (16 seizure-free), and 21 healthy matched controls performed an exhaustive bicycle ergometer test. Immediately before and after the exercise test, resting state electroencephalography measurements (Brain Products GmbH, 128-channel actiCHamp) of 5 min were carried out to investigate functional connectivity assessed by phase locking value in source space for whole brain, central autonomic network and visual network. Additionally, 1-lead ECG (Brain products GmbH) was performed to analyze parasympathetic (root mean square of successive differences (RMSSD) of the heart rate variability) and sympathetic activity (electrodermal activity (meanEDA)). MeanEDA increased (*p* < 0.001) and RMSSD decreased (*p* < 0.001) from pre to post-exercise in both groups. Correlation coefficients of meanEDA and central autonomic network functional connectivity differed significantly between the groups (*p* = 0.004) after exercise. Both patients with epilepsy and normal control subjects revealed the expected physiological peripheral autonomic responses to acute exhaustive exercise, but alterations of the correlation between central autonomic and peripheral sympathetic activity may indicate a different sympathetic reactivity after exercise in patients with epilepsy. The clinical relevance of this finding and its modulators (seizures, anti-seizure medication, etc.) still needs to be elucidated.

**Supplementary Information:**

The online version contains supplementary material available at 10.1007/s00221-024-06792-0.

## Introduction

Sympathetic activation and parasympathetic deactivation are typical responses of the autonomic nervous system (ANS) to exhaustive exercise. While these cardiovascular and ventilatory adaptations are mainly driven by the central autonomic network (CAN) (Fu and Levine 2013), peripherally obtained ANS parameters like heart rate variability (HRV) or electrodermal activity (EDA) allow an objective assessment of distinct sympathetic (EDA) and parasympathetic (root mean square of successive heart beat differences (RMSSD)) activity. Consequently, EDA increases (Posada-Quintero and Chon 2020) and RMSSD decreases during exercise (Shaffer et al. 2014).

In epilepsy, one of the most prevalent neurological diseases (Fiest et al. 2017), sympathetic/parasympathetic balance within the ANS is shifted towards sympathetic activation (Chroni et al. 2009; Mativo et al. 2010; Poh et al. 2012) both during and between seizures (Goit et al. 2016; Horinouchi et al. 2019; Lotufo et al. 2012; Poh et al. 2012; Sarkis et al. 2015; Vieluf et al. 2021). Although this change is most likely driven by CAN activation, data on direct CAN activity in relation to exercise is scarce. Epilepsy-induced centrally mediated dysautonomia, however, is well described in epilepsy and in its most severe form even associated with sudden explained death in epilepsy (SUDEP) (Devinsky 2004). But studies investigating autonomic dysfunction in patients with epilepsy (PWE), are mostly performed on patients with therapy refractory epilepsy, the majority of PWE might be underrepresented in ANS research, since most PWE have well-controlled seizures (Kwan and Brodie 2000).

As exercise tests have been shown to feasibly detect even subtle pathological changes of physiological responses within the cardiovascular system (Ashley et al. 2000; Marcadet et al. 2018), it is used in this study to examine differential ANS network activity in PWE. As peripheral ANS parameters after exercise do not differ between PWE and healthy control subjects, but in chronotropic competence in response to exercise (van den Bongard et al. 2022). Therefore, no differences in EDA and RMSSD were expected between PWE and healthy controls, but it was hypothesized that CAN response and the interrelation between the CAN with peripheral ANS parameters after exhausting exercise might be altered in PWE.

## Methods

This study was registered at the German Clinical Trail Register (DRKS00014822) and was conducted in accordance with the Declaration of Helsinki. The study protocol and the informed consent, obtained by each participant before enrolment, were approved by the ethics committee of the Westfalian Medical Board. Recruitment and measurements took place between July 2018 and December 2021.

Recruitment and in- and exclusion criteria have been described elsewhere (van den Bongard et al. 2022). In brief, people with diagnosed epilepsy (all syndromes) and healthy controls, matched by age (18–60 years old), sex and Body Mass Index (BMI), without physical impairments, severe cardiovascular diseases or brain lesions were included.

### Exercise test

All participants conducted an exhaustive exercise test on a bicycle ergometer (Excalibur, Lode), consisting of a 2-min warm up at 24 watts (W), an incremental load increase (12 W per minute) until exhaustion and a 2-min cool down at 24 W. Revolutions per minute (rpm) were based on the load (W): 50–60 rpm at 24–60 W, 60–70 rpm at 60–100 W, 70–70 rpm at > 100 W. Spiroergometry (Metalyzer 3B, Cortex) was carried out during the exercise test.

### Measurements

5-min supine resting state measurements were conducted immediately before and after (start of the measurements 658 ± 98.7 sec. post-exercise) the exhaustive exercise test in a relaxed, awake state with eyes closed, consisting of the recording of an electroencephalogram (EEG) and ANS parameters (RMSSD, meanEDA).

### ANS recordings, analysis and outcomes

1-lead electrocardiogram (ECG) (Brain Products GmbH) and a Galvanic Skin Response Module (GSR) (Brain Products GmbH) were used to record ANS signals. Sampling frequency for both devices was at 1000 Hz. RMSSD, a parameter indicating parasympathetic activity of HRV as well as meanEDA indicating sympathetic activity were measured (Posada-Quintero and Chon 2020).

To calculate RMSSD, ECG was down sampled to 250 Hz to reduce data points and a Zero Phase Shift Butterworth Filter (low cutoff 8 Hz, time constant[s] 0.1591549, Order 4; high cutoff 20 Hz, Order 4) was applied to reduce noise (Fedotov 2016) (BrainVision Analyzer 2.1.2, Brain Products GmbH). ECG Marker solution, implemented in BrainVision Analyzer, was used for R-peak detection. RR-intervals of the 5-min recording were calculated and exported to Kubios® (Kubios ® HRV Standard 3.1.0.). Threshold-based artifact correction with a medium threshold was used to detect and correct artifacts (Kaufmann et al. 2011). Based on the clean data, RMSSD was calculated over the 5-min window (Task Force of The European Society of Cardiology and The North American 1996).

For EDA recordings, two electrodes (GSR Module) were placed on the middle phalanges of the index and middle finger on the non-dominant hand. Data were down sampled to 250 Hz to reduce data points (BrainVision Analyzer 2.1.2, Brain Products GmbH). A moving average (window size 91 points) (Vieluf et al. 2019), implemented as solution in the BrainVision Analyzer, was applied. Data were exported to Excel and meanEDA was calculated over the 5-min window. The size of analysis window was selected to match the HRV analysis window.

### EEG recording, analysis and outcomes

A 128-channel EEG was used (actiCHamp, Brain Products GmbH). The EEG cap was placed on the head according to the 10–10 system. The sampling rate was 1000 Hz. FPz (frontopolar midline electrode) was utilized as ground electrode and the reference electrode was FCz (frontocentral midline electrode). Impedances were kept below 25 kΩ. For preprocessing, data were down sampled to 250 Hz to reduce data points (van Diessen et al. 2015). Data sets were checked for electrode bridges by using the Matlab-based eBridge Algorithm (Alschuler et al. 2014) and the magnitude-squared coherence in BrainVision Analyzer (BrainVision Analyzer 2.1.2, Brain Products GmbH). For the latter, coherences above 0.9 were defined as electrode bridges. Electrically bridged channels were interpolated by Topographic Interpolation by Spherical Splines when detected by both, Matlab-based eBridge Algorithm and magnitude-squared coherence (Goelz et al. 2021). After bridge check, a Zero Phase Shift Butterworth Filter with a low cutoff of 1 Hz (time constant [s]: 0.1591549, Order 4) and a high cutoff of 30 Hz (Order 4) as well as a notch filter (50 Hz) were applied. Topographic Interpolation by Spherical Splines were used for noisy channels which were identified via visual inspection. An average reference was used (Zheng et al. 2018) and individual electrode positions, recorded by CapTrak (Brain Products GmbH), were loaded. Independent Component Analysis (ICA, infomax) was used for artifact correction (eye, ECG). After that, remaining artifacts were manually marked and the first 4 artifact free segments with a window size of 8.192 s per segment (Engels et al. 2015) were exported for further analysis to BrainStorm (Version: 3.220222) (Tadel et al. 2011). Segments were visually inspected and clinically evaluated by a board certified epileptologist (CR) prior to further connectivity analysis.

Matlab-based BrainStorm software (Version of March 2021) was used for connectivity analysis. A default anatomy, Colin27 template was used (Rizkallah et al. 2020). Individual EEG electrode positions obtained via CapTrak were used to warp the template to approximate the individual head shape. Subsequently, an identity matrix (no noise modelling) was used. The Boundary Element Method (BEM) was used to calculate the head model (Gramfort et al. 2010). If a dipole error appeared, dipoles were forced inside the skull (maximum number of forced dipoles 4). Source estimation was done by minimum norm imaging. Functional connectivity was calculated by phase locking value (PLV) based on the Desikan-Killiany Atlas per segment. After that, connectivity matrices of all four segments were averaged (Samogin et al. 2020). The averaged connectivity matrix was exported for the alpha frequency band (8–12 Hz). MeanPLV was calculated over 68 regions of interest (ROI) for whole brain analysis and over 24 ROI of the CAN (Beissner et al. 2013). The visual network (VIS, 7 ROI) was used as a reference network (Kabbara et al. 2017), because the visual system may not be strained or otherwise specifically affected by ergometry.

### Statistics

Data were tested for normality by Shapiro–Wilk Test (*p* > 0.05) and for equal variances by Levene-Test (*p* > 0.05). Within-group differences were determined by Student’s t-test or Wilcoxon rank sum test. Between-group differences were assessed by Kruskal–Wallis test since requirements for parametric testing were not fulfilled, except for the relative VO2max, where a Student’s t-test was used. Effect sizes were assessed by Person’s correlation coefficient (r). Interrelations were determined by Spearman Rank correlation coefficients. Differences between correlations coefficients were calculated by Fisher’s z-transformation (Ramseyer 1979). Partial correlation was used to assess the influence of control variables on correlations. The level of significance was determined as < 0.05. To control for multiple testing, Bonferroni method was used (Lee and Lee 2018). However, due to the explorative nature of this pilot study, both corrected and uncorrected *p*-values were reported and no sample size calculation could be performed. For statistical analysis, IBM SPSS Statistics (Version 25.0.0.1) was used.

## Results

### Subjects

21 PWE (39 ± 11.6 yrs., BMI 26.7 ± 4.2, female *n* = 12, male *n* = 9) and 21 healthy matched controls (38.04 ± 11.5 yrs., BMI 27.1 ± 5.2, female *n* = 12, male = 9) participated in the study. No differences in clinical characteristics were detected except for alcohol use with a higher consumption in the control group (*p* = 0.01) (Table 1).

**Table 1:** Clinical characteristics of epilepsy and control group

Variables	Patients (*n* = 21)	Control (*n* = 21)	*p*
Age (yrs.)	39 ± 11.6	38.04 ± 11.5	^c^0.791
BMI	26.7 ± 4.2	27.1 ± 5.2	^c^0.772
Sex	*f* = 12; *m* = 9	f = 12; m = 9	
Hypertension	2	0	^b^0.488
Diabetes type 1	1	0	^b^1.00
Dyslipidaemia	1	1	^b^1.00
Active smoking	3	1	^b^0.606
Active smoking in the past	4	6	^a^0.469
Alcohol use	12	19	^a^0.014*
Family history CVD	10	8	^a^0.533
Family history epilepsy	2	3	^b^1.00

^a^Chi squared test

^b^Fisher‘s exact test

^c^Students t-test

^*^p < 0.05

^**^p < 0.01

Age, BMI: mean ± standard deviation

Further characteristics: n

CVD = cardiovascular disease, f = female, m = male

Mean duration of epilepsy was 18.83 ± 19.06 years (Table 2). PWE suffered from different types of seizures. Most of the patients were seizure-free for at least 6 months (*n* = 16). Three patients had 1–2 seizures per month, one patient had one seizure per month and one patient had one seizure per week. For seizure-free patients, mean time to the last seizure was 3145.3 ± 3264.7 days. 14 patients were under anti-seizure drug (ASD) monotherapy. Five patients took two ASD, one patient took three ASD and one patient was not under ASD medication (lamotrigine (*n* = 12), levetiracetam (*n* = 4), valproic acid (*n* = 6), lacosamide (*n* = 1), carbamazepine (*n* = 3), ethosuximide (*n* = 1)). Detailed information about dosages and serum levels can be found in Table S1.

**Table 2:** Characterization of seizures and epilepsy

Variables	Patients (*n* = 21)
Disease duration (yrs.)	18.83 ± 19.06
Age of disease onset	20.19 ± 14.08
Seizure type* Generalized motor Generalized nonmotor Focal awareness impaired Focal aware Unknown type	10 3 4 3 4
Seizure frequency (last 6 months) Seizure free 1–2 1 per month 1 per week	16 3 1 1
Last seizure of seizure-free patients (*n* = 16) (days)	3145.3 ± 3264.7 (min: 356; max: 9490)
Number of ASD 0 1 2 3	1 14 5 1

^*^Three patients indicated two types of seizures

Disease duration, age of disease onset, last seizure of seizure-free patients: mean ± standard deviation

Adverse events before, during or after the exhaustive exercise test were not observed in either group. Each participant achieved individual exhaustion based on ventilatory, metabolic, cardiovascular and performance criteria. There was difference between epilepsy and control group regarding cardiorespiratory fitness, assessed by relative VO_2_ max (31.04 ± 8.88 vs 33.42 ± 7.40, *t* = −0.949, df = 40, *p* = 0.348) (Table 3).

**Table 3:** Peripheral autonomic activity and functional connectivity before and after exercise

				Patients (*n* = 21)		Controls (*n* = 21)		
				Mean	SD	Mean	SD	p (ES (r))
	RMSSD pre (ms)			55.44	53.84	42.48	30.22	^c^0.71
	RMSSD post			13.54	15.86	8.79	6.56	^c^0.37
		RMSSD pre vs. post	p ES (r)	^b^ < 0.001**^1^ 0.770		^b^ < 0.001**^1^ 0.868		
	meanEDA pre (µS)			1.36	0.85	1.68	1.10	^c^0.32
	meanEDA post			2.99	1.35	2.65	1.68	^c^0.26
		meanEDA pre vs. post	p ES (r)	^b^ < 0.001**^1^ 0.868		^b^ < 0.001**^1^ 0.793		
Whole brain	PLV alpha pre			0.30	0.09	0.28	0.04	^c^0.95
	PLV alpha post			0.32	0.06	0.30	0.05	^c^0.34
		PLV pre vs. post	p ES (r)	^b^0.04* 0.447		^a^0.006**^1^ 0.564		
CAN	PLV alpha pre			0.33	0.09	0.31	0.05	^c^0.78
	PLV alpha post			0.35	0.07	0.33	0.05	^c^0.42
		PLV pre vs. post	p ES (r)	^b^0.14 0.318		^b^0.03* 0.474		
VIS	PLV alpha pre			0.42	0.08	0.42	0.04	^c^0.42
	PLV alpha post			0.44	0.06	0.43	0.05	^c^0.58
		PLV pre vs. post	p ES (r)	^b^0.08 0.371		^b^0.82 0.049		
	relative VO_2_max			31.04	8.88	33.43	7.40	^a^0.34 (0.148)

^a^t-Test

^b^Wilcoxon rank sum

^c^Kruskal Wallis test

^*^p < 0.05 (uncorrected)

^**^p < 0.01 (uncorrected)

^1^Significant after post hoc Bonferroni correction

alpha 8–12 Hz

*ES* effect size, *PLV* phase locking value, *CAN* central autonomic network, *VIS* visual network, *EDA* electrodermal activity, *RMSSD* Root mean square of the successive differences

### Functional connectivity

Before post hoc correction, whole brain PLV increased in both the epilepsy group (0.30 ± 0.09 vs. 0.32 ± 0.06) and control group (0.28 ± 0.04 vs. 0.30 ± 0.05, *t* = −3.061, df = 20, p = 0.006) from pre- to post-exercise. This relationship stayed significant after post hoc Bonferroni correction in the control group only. CAN PLV from pre- to post-exercise increased significantly in the control group only (0.31 ± 0.05 vs 0.33 ± 0.05, *z* = −2.173, *p* = 0.03), but significance did not stay after the correction for multiple comparisons.

No significant changes from pre- to post-exercise were observed for VIS PLV for either group and there were no group differences within the measurement time points for whole brain, CAN and VIS PLV (Table 3).

### Peripheral autonomic responses

RMSSD decreased significantly from pre- to post-exercise in the epilepsy group (55.44 ± 53.84 vs. 13.54 ± 15.86, *z* = −5.277, *p* < 0.001) and in the control group (42.48 ± 30.22 vs. 8.79 ± 6.56, *z* = −3.980, *p* < 0.001). No between group differences pre- and post-exercise were detected (Table 3).

MeanEDA increased significantly from pre- to post-exercise in the epilepsy group (1.36 ± 0.85 vs. 2.99 ± 1.35, *z* = −0.408, *p* < 0.001) as well as in the control group (1.68 ± 1.10 vs. 2.65 ± 1.68, *z* = −3.632, *p* < 0.001). No between group differences pre- and post-exercise were detected (Table 3).

### Correlation analysis

After post hoc correction for multiple testing no significant correlations between CAN PLV or VIS PLV with meanEDA or RMSSD could be detected.

However, before correction for multiple comparisons, CAN PLV correlated significantly with meanEDA (*r* = 0.543, *p* = 0.011) post-exercise in the control group (Table 4).

**Table 4:** Correlation coefficients of peripheral autonomic activity und functional connectivity before and after exercise

			Pre-exercise				Post-exercise			
			meanEDA		RMSSD		meanEDA		RMSSD	
			Epilepsy	Control	Epilepsy	Control	Epilepsy	Control	Epilepsy	Control
alpha 8–12 Hz	CAN PLV	*r*	0.376	0.080	−0.380	−0.184	−0.280	0.543	−0.429	−0.133
		*p*	0.093	0.731	0.089	0.423	0.219	0.011*	0.052	0.567
	VIS PLV	*r*	0.216	0.069	−0.352	0.118	0.071	−0.166	−0.161	0.299
		*p*	0.346	0.767	0.117	0.610	0.758	0.471	0.485	0.188

^*^p < 0.05 (uncorrected)

^**^p < 0.01 (uncorrected)

^1^Significant after post hoc Bonferroni correction

r = Spearman Rank correlation coefficient

*PLV* phase locking value, *CAN* central autonomic network, *VIS* visual network, *EDA* electrodermal activity, *RMSSD* Root mean square of the successive differences

**Fig. 1:**
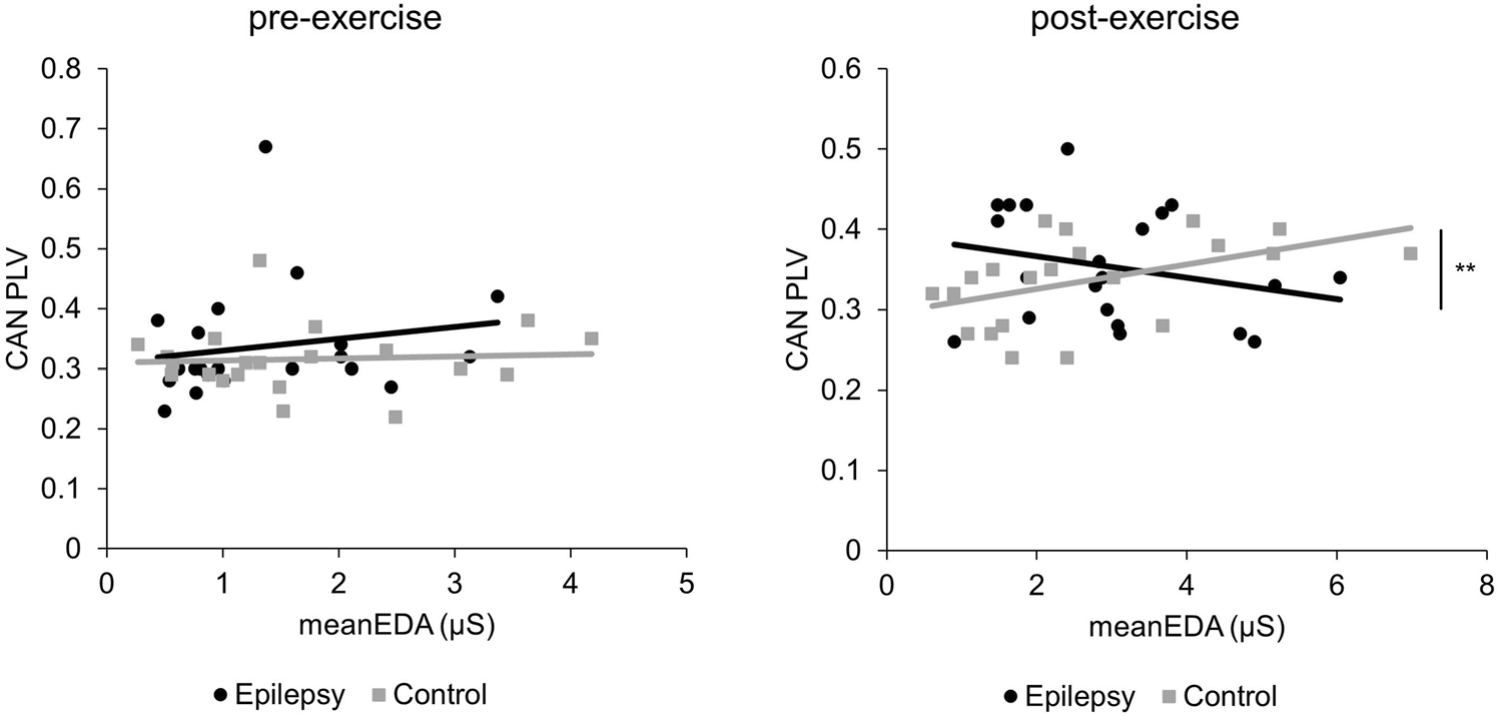
Correlations of CAN functional connectivity and meanEDA before and after exercise. *PLV* phase locking value, *CAN* central autonomic network, *EDA* electrodermal activity, *p < 0.05, **p < 0.01

Correlation coefficients of CAN PLV and meanEDA after exercise differed significantly between the groups (*p* = 0.004) (Table 5) (Fig. 1).

**Table 5:** Group comparisons (epilepsy vs. control group) of the correlation coefficients of peripheral autonomic activity and functional connectivity

			meanEDA	RMSSD		meanEDA	RMSSD
Pre-exercise	alpha 8–12 Hz		p	p	Post-exercise	p	p
		CAN PLV	0.172	0.26		0.004**^1^	0.165
		VIS PLV	0.326	0.072		0.237	0.079

^*^p < 0.05 (uncorrected)

^**^p < 0.01 (uncorrected)

^1^Significant after post hoc Bonferroni correction

*PLV* phase locking value, *CAN* central autonomic network, *VIS* visual network, *EDA* electrodermal activity, *RMSSD* Root mean square of the successive differences

### Control variables

Since the subgroup of patients who took lamotrigine was considerably larger, the influence of lamotrigine on CAN PLV and meanEDA was assessed. No significant influence was detected (*r* = −0.192, *p* = 0.471).

There was no significant influence of the relative VO_2_ max on CAN PLV and meanEDA as well as on CAN PLV and RMSSD correlations post-exercise (Table S2).

## Discussion

The investigation of central and peripheral ANS function before and after an acute bout of exhaustive exercise revealed no group difference in whole brain and CAN functional connectivity. As expected, sympathetic activity (meanEDA) increased and parasympathetic activity (RMSSD) decreased significantly after acute exercise in the epilepsy and the control group. However, the connection between CAN functional connectivity and meanEDA, expressed by the correlation coefficient, differed significantly between both groups after exercise.

Both groups revealed a similar increase in whole brain and CAN functional connectivity after exercise. This is in line with previous reports in the literature since bouts of acute exercise have been shown to increase functional brain connectivity (Moore et al. 2022). The lack of a group difference could be caused by the composition of the epilepsy group mainly consisting of PWE with controlled seizures, as it is known that, amongst others, seizure frequency is a potential modifier that might impact altered brain network function (van Diessen et al. 2013).

Acute exercise is usually associated with increased sympathetic and decreased parasympathetic activation (Gladwell et al. 2010; Vieluf et al. 2019) and those changes often persist post-exercise (Gladwell et al. 2010). Both groups exhibited this pattern as revealed by increased meanEDA and decreased RMSSD after exercise. No group differences could be observed in both measurement time points. High standard deviations confirm the individuality of autonomic activity (Garet et al. 2004), but the lack of a group difference between the investigated cohort of PWE and healthy controls might also be driven by the high proportion of reasonably well-controlled PWE, who are known to more rarely exhibit autonomic dysfunction when compared to therapy refractory PWE (Ansakorpi et al. 2000). Moreover, ASDs could influence autonomic activity. Higher dosages of Na-blocker ASD, like lamotrigine or carbamazepine, might decrease heart rate (Thijs et al. 2021). Additionally, previous studies investigating influences of ASDs or ASD withdrawal (Kennebäck et al. 1997; Lossius et al. 2007) as well as the influence of a lack of ASD medication (Persson et al. 2007) confirmed at least a partial influence of ASD medication on autonomic alterations, potentially due to the influence on ion channels (Chindo et al. 2016).

Compared to the control group, the connection between central and peripheral sympathetic control in PWE seems to be different after exercise, indicated by significantly different correlation coefficients of CAN functional connectivity and meanEDA. This observation appears to be specific for the CAN because no group difference was observed for the VIS network that served as a reference network. The CAN is a widespread network connecting cortical, subcortical regions (Beissner et al. 2013) and the brain stem (Sklerov et al. 2019) and modulates the ANS in resting condition as well as in response to certain stimuli (Sklerov et al. 2019). During exercise, the sympatho-excitatory response is modulated by a central command that is, under healthy conditions, also associated with parasympathetic withdrawal (Bishop 2004). The induction of increased sympathetic activity during the stress situation of an exercise test (Freeman et al. 2006) is confirmed by an increase in meanEDA and in PWE not different from normal control subjects.

An increased sympathetic tone between seizures is a well-known phenomenon in epilepsy (Lotufo et al. 2012; Myers et al. 2018; Romigi et al. 2016; Sevcencu and Struijk 2010) and has mostly been demonstrated in patients with therapy refractory seizures. In contrast, our cohort consists of a large proportion of seizure-free patients and may, therefore, rather be representative for PWE in general (Kwan and Brodie 2000). Consequently, it could be argued that the central sympathetic control may not differ to a large extent from healthy controls. Furthermore, the investigations of this study focused on an acute exercise test and it was already shown that performance and fitness parameters did not differ between PWE and controls (van den Bongard et al. 2022). Nevertheless, the connection between central control and peripheral sympathetic activity seems to be different in PWE compared to controls after exercise. Therefore, factors in PWE independent from seizure control have to be considered that might contribute to central and peripheral autonomic alterations. For instance, medications like ASD, as described before, or beta blockers influence autonomic function (Thijs et al. 2021). Additionally, ASD may impact central network activity in general. Carbamazepine was shown to change brain graph topology (Haneef et al. 2015) and levetiracetam treatment was associated with inter- and intranetwork alterations (Pang et al. 2020). Although patients took different ASDs, subgroup analysis for all ASD subgroups was not possible due to small sample sizes. However, a larger group taking lamotrigine (12/21) were examined and did not demonstrate differences in CAN functional connectivity and meanEDA, correlations. Besides the ASD influence, the clinical epilepsy syndrome might impact autonomic control. Allen et al. 2017 observed abnormalities in brain regions involved in autonomic processes in patients with epilepsy at high risk for sudden unexpected death. The syndromal heterogeneity of our cohort prohibited further statistical analysis, but the variety of clinical syndromes could certainly have influenced sympathetic control (Shaker et al. 2021; Thijs 2019).

After exercise, a significant correlation before post hoc correction between CAN functional connectivity and meanEDA in the control group might indicate a stronger functional sympathetic central–peripheral connection and therefore the basis for the exercise-induced increase in sympathetic activity. This correlation did not become apparent in the epilepsy group, possibly related to different (and less controlled) functional sympathetic activity in PWE. A larger sample size might be able to elucidate this hypothesis further.

In this study, a multidimensional approach was used for investigating the autonomic system in PWE extending previous research focusing on either the central (for example utilizing fMRI (Sklerov et al. 2019)) or the peripheral part (for example utilizing parameters of the HRV (Lotufo et al. 2012; Mativo et al. 2010)). Assessment of ANS subsystems individually and in relation to each other adds important information to the investigation of alterations in the ANS (Vieluf et al. 2019). The results of this study indicate a difference in the interaction of central autonomic and peripheral sympathetic activity after an exhaustive exercise test between PWE and healthy controls. This might be explained by the impact on brain networks in PWE (van Diessen et al. 2013) despite the relative heterogeneity of epilepsy syndromes and the relative low seizure burden in the subjects of our study. The previously described increased sympathetic tone in PWE might also be induced by these autonomic network alterations (Myers et al. 2018). Exercise might be used in PWE as a stressor to investigate ANS alterations. In addition, it will be interesting how those might also be modified by training, which is traditionally used to target psychological comorbidities and seizures in PWE. In the future, exercise tests in conjunction with autonomic measures might be used as a diagnostic tool to differentiate PWE from healthy people. Although connectivity measures have to be further explored, the presented approach may extend the common visual and automated digital interpretations of clinical EEGs, but requires a larger number of electrodes than clinical routine EEGs. If future studies may elucidate further, how exercise tests can be used to demarcate central–peripheral ANS alterations in PWE and if and how those alterations might be modified by chronic exercise, exercise may hypothetically also affect the risk for life-threatening conditions of PWE like sudden unexpected death in epilepsy (SUDEP), which occurs as the most dramatic form of dysautonomia, in a positive way.

### Limitations

Several limitations must be considered. Only a small number of epilepsy patients with different types of seizures and different seizure frequencies were included. Although most of the patients were seizure-free, some of them still had uncontrolled seizures. Nevertheless, this study sample represents a community-based group of epilepsy patients, where approximately 70% are seizure-free (Kwan and Brodie 2000). PWE took different ASD, with different dosages and serum levels, preventing further statistical exploration of significant ASD effects. Methodologically, not all CAN regions can be assessed by EEG. ROIs were predominantly included in cortical regions since deeper structures may not contribute significantly to surface EEG signals. Source reconstruction itself bears some inaccuracies, but utilization of 128-channel EEG provides the highest temporal resolution. The combination with an MRI template and warping its surface by individual electrode position was associated with increased spatial resolution as compared to EEG alone, but using individual anatomical models would even be more accurate.

## Conclusion

PWE reveal similar peripheral autonomic reactions to an acute bout of exercise in comparison to normal control subjects, but exhibit a different connection between central autonomic and peripheral sympathetic activity. Since the majority of PWE were seizure-free, the mechanisms contributing to this finding remain unclear. Further insight into modifiers, clinical consequences and potential response to chronic exercise and training may guide future therapeutic interventions more effectively.

### Supplementary Information

Below is the link to the electronic supplementary material.

Supplementary file1 (DOCX 17 KB)

## Data Availability

The data that support the findings of this study are not publicly available because it contains medical information from medical records and are available from the corresponding author upon reasonable request. Data are located in controlled access data storage at the Institute of Sports Medicine (Paderborn University).

## Abbreviations

PWE: Patients with epilepsy
ANS: Autonomic nervous system
HRV: Heart rate variability
RMSSD: Root mean square of successive differences
EDA: Electrodermal activity
PLV: Phase locking value
EEG: Electroencephalography
CAN: Central autonomic network
VIS: Visual network
ASD: Anti-seizure drug
