# Supplementary material for: Exercise-induced central and peripheral sympathetic activity in a community-based group of epilepsy patients differ from healthy controls
Source: Exp Brain Res. 2024 Mar 29;242(6):1301–10. doi: 10.1007/s00221-024-06792-0 (PMC11108887; doi:10.1007/s00221-024-06792-0)
Supplement: Supplementary file 1 — Supplementary file1 (DOCX 17 KB) [file 221_2024_6792_MOESM1_ESM.docx]

**Supplement**

**Table S1**

ASD daily dosage and serum level

| **ASD** |  | **dose (mg)** | | | | |  |  |  |  |
| --- | --- | --- | --- | --- | --- | --- | --- | --- | --- | --- |
|  | **n** | **median** | **min** | **max** | **25% percentile** | **75% percentile** |  |  |  |  |
| Lamotrigine | 12 | 250 | 100 | 400 | 168.75 | 100 |  |  |  |  |
| Levetiracetam | 4 | 2250 | 1500 | 3000 | 1500 | 1500 |  |  |  |  |
| Valproic acid | 6 | 1250 | 600 | 2750 | 600 | 600 |  |  |  |  |
| Lacosamide | 1 | 200 | 200 | 200 | 200 | 200 |  |  |  |  |
| Carbamazepine | 3 | 600 | 400 | 800 | 400 | 400 |  |  |  |  |
| Ethosuximide* | 1 | 250 | 250 | 250 | 250 | 250 |  |  |  |  |
|  |  | **serum level (mg/l)** | | | | | | **time ASD intake and blood sample (min)** | | |
|  |  | **median** | **min** | **max** | **25% percentile** | **75% percentile** | **reference range** | **median** | **min** | **max** |
| Lamotrigine |  | 4.85 | 1.90 | 10.30 | 3.27 | 1.9 | 3-14 | 262.5 | 70 | 491 |
| Levetiracetam |  | 27.50 | 14.80 | 45.90 | 14.8 | 14.8 | 10-40 | 371 | 203 | 600 |
| Valproic acid |  | 50.50 | 30 | 102 | 30 | 59.5 | 40-100 | 420 | 100 | 1063 |
| Lacosamide |  | 0.60 | 0.60 | 0.60 | 0.6 | 0.6 | 1-10 | 491 | 491 | 491 |
| Carbamazepine |  | 7.20 | 3.60 | 9.30 | 3.6 | 3.6 | 4-12 | 360 | 100 | 502 |

* serum level of ethosuximide analysis was not determined

**Table S2**

Partial correlation post exercise

|  | | CAN PLV and meanEDA (Pearson correlation) | CAN PLV and meanEDA controlled for relative VO_2_max | CAN PLV and RMSSD (Pearson correlation) | CAN PLV and RMSSD controlled for relative VO_2_max |
| --- | --- | --- | --- | --- | --- |
| epilepsy | r | -0.268 | -0.273 | -0.324 | -0.317 |
|  | p | 0.240 | 0.244 | 0.153 | 0.173 |
| control | r | 0.467 | 0.456 | -0.229 | -0.127 |
|  | p | 0.033* | 0.043* | 0.318 | 0.593 |

* p<0.05
